# Association between vision-specific quality of life and falls in community-dwelling older adults: LOHAS

**DOI:** 10.1371/journal.pone.0195806

**Published:** 2018-04-24

**Authors:** Kakuya Niihata, Shingo Fukuma, Yoshimune Hiratsuka, Koichi Ono, Masakazu Yamada, Miho Sekiguchi, Koji Otani, Shinichi Kikuchi, Shinichi Konno, Shunichi Fukuhara

**Affiliations:** 1 Division of Clinical Epidemiology, Fukushima Medical University, Fukushima, Japan; 2 Center for Innovative Research for Communities and Clinical Excellence (CiRC^2^LE), Fukushima Medical University, Fukushima, Japan; 3 Department of Healthcare Epidemiology, School of Public Health in the Graduate School of Medicine, Kyoto University, Kyoto, Japan; 4 Institute for Health Outcomes and Process Evaluation Research (iHope International), Kyoto, Japan; 5 Human Health Sciences, Graduate School of Medicine, Kyoto University, Kyoto, Japan; 6 Department of Ophthalmology, Juntendo University Graduate School of Medicine, Tokyo, Japan; 7 Department of Ophthalmology, Juntendo Tokyo Koto Geriatric Medical Center, Tokyo, Japan; 8 Department of Ophthalmology, Kyorin University, Tokyo, Japan; 9 Department of Orthopedic Surgery, School Medicine, Fukushima Medical University, Fukushima, Japan; 10 Fukushima Global Medical Science Center, Fukushima Medical University, Fukushima, Japan; George Institute for Global Health, AUSTRALIA

## Abstract

**Background:**

Falls and fall-related fractures are a major public health problem among the older adults. Although objective measures of poor vision have been reported to be associated with falls, the association of self-reported visual function and vision-specific quality of life (QOL) with falls has been inconsistent across several studies. We investigated the association of self-reported visual function and vision specific QOL with falls in community-dwelling older adults.

**Methods:**

We conducted a cross-sectional analysis using the baseline data from participants of the Locomotive Syndrome and Health Outcome in Aizu Cohort Study (LOHAS), which is an ongoing population-based cohort study to evaluate the association of physical dysfunction with the clinical outcomes in community-dwelling people. In the present study, the participants aged over 65 years in 2010 were eligible. The exposure variable was the composite score of the VFQ-J11, which was newly developed using item response theory to evaluate vision specific QOL, and the self-reported outcomes were any fall and frequent falls (≥2) over a 1-month period. We estimated odds ratios using separate logistic regression models adjusted for relevant confounding factors.

**Results:**

Among 1624 participants, the median (interquartile range) composite score of VFQ-J11 was 86.8 (76.0–95.9). Any fall and frequent falls were reported by 13.9% and 5.4% of participants, respectively. The composite score of the VFQ-J11 was significantly associated with both frequent falls (adjusted ORs per 10 points, 0.80; 95% CI, 0.68–0.93) and any fall (adjusted ORs per 10 points, 0.84; 95% CI, 0.76–0.94).

**Conclusions:**

We found that the composite score of the VFQ-J11 was associated with falls in community-dwelling older adults. Detecting individuals with visual impairments associated with falls using the VFQ-J11 and improvement in the score by interventions could prevent falls. We may consider adding self-reported visual function and vision-specific QOL to conventional risk factors for fall among older adults.

## Introduction

Falls and fall-related fractures are a major public health problem, especially among older adults. Reportedly, 30% of community-dwelling older adults older than 65 years fall at least once annually, with 5% of falls resulting in fractures and 10% of falls resulting in other serious injury[1,2]. While there are multiple risk factors for falls in older adults, vision impairment is considered one of the important factors contributing to the risk of falls[3]. Several aspects of visual function, such as visual acuity, contrast sensitivity, and visual fields, have been reportedly associated with falls[4–7]. These reports suggest that detecting individuals with visual impairments associated with falls and modifying these impairments are important to preventing falls in older adults.

Although self-reporting of visual dysfunction might be a useful means of detecting affected individuals, the association between self-reported visual function and falls has been inconsistent across several studies, with several studies reporting a significant association between visual impairment and falls[8,9] and others showing no significant association[10,11]. Several factors could contribute to this inconsistency. First, the confounding factors adjusted in the studies varied. In those studies showing significant associations, the results came from the crude analysis[9] or the risk prediction model, not from fully adjusted analyses that accounted for all potential confounding factors[8]. Second, the questionnaires used to investigate visual impairment varied among the different aspects of vision, such as distant vision and near vision. Moreover, these questionnaires have not been validated.

The National Eye Institute Visual Function Questionnaire (NEI-VFQ) was developed to measure vision-targeted quality of life (QOL) on the basis that objective clinical measures, such as visual acuity and visual field, cannot fully capture how activities of daily living are affected by vision impairment[12]. Suzukamo et al. developed a Japanese version of the NEI-VFQ, which comprises 25 items (VFQ25)[12], while Fukuhara et al. recently developed a short version from VFQ25, the Visual Function Questionnaire 11-item Japanese Version (VFQ-J11), which comprises 11 items identified using item response theory[13]. Although the association between the VFQ25 and falls has been reported[14], this study did not adjust for relevant confounding factors and limited its univariate analysis to the association of subscales of the VFQ25 with falls. Accordingly, the association of vision-specific QOL with falls remains unclear. Although the VFQ-J11 is reportedly superior to the VFQ25 in terms of responsiveness and criterion-related validity with visual acuity and field of vision[13], it is still unclear how VFQ-J11 scores are relevant to clinical outcomes, such as falls. It has been reported that VFQ-J11 score was significantly improved by cataract surgery[15], with a change in VFQ-J11 score (standard deviation [SD]) to 15.53 (16.52) after the 1^st^ eye surgery, 12.97 (15.37) after the 2^nd^ eye surgery, and 22.16 (19.11) after surgery in both eyes. However, it is also unclear how these changes in VFQ-J11 score affect the risk of falls in individual subjects.

Here, we investigated the association between the VFQ-J11 and falls in a cross-sectional study. We evaluated how VFQ-J11 scores are related to falls and how useful the VFQ-J11 is in detecting individuals with visual impairment associated with falls.

## Subjects and methods

### Design and setting

The Locomotive Syndrome and Health Outcomes in Aizu Cohort Study (LOHAS) is an ongoing population-based cohort study which is evaluating the association of physical dysfunction with the clinical outcomes, such as cardiovascular disease, QOL, medical costs, and mortality. In LOHAS, physical function and health-related QOL, including not only objective metrics by physical examinations but subjective metrics using self-administered questionnaires were measured at the baseline evaluation between 2008 and 2010, which was linked with annual health check-ups conducted in the local municipalities. Especially, the unique measurement items including symptoms of overactive bladder and vision-specific QOL, which had not been installed in annual health check-ups, were measured in 2010. The participants of the LOHAS comprised the residents of two municipalities in Fukushima Prefecture, Japan, aged over 40 years, receiving regular health check-ups conducted by local government annually. There were no exclusion criteria. The participants were planned for follow-up of 10 years, linked with the administrative data offered by the municipalities, including the medical receipts and death certificate, to evaluate the clinical relevant outcomes. The design of the LOHAS has been described in detail elsewhere [16]. In the present study, we conducted a cross-sectional analysis using data from participants of LOHAS in 2010. Written informed consent to analyze data collected in LOHAS was obtained from all participants on enrollment. The study complied with the Declaration of Helsinki and was approved by the Research Ethics Committee of Fukushima Medical University School of Medicine.

### Study population

In the present study, eligible participants were aged ≥65 years old. Participants with missing exposure and outcome data were excluded from the final analyses. Missing exposure was defined as not answering all items in the VFQ-J11.

### Exposure

The main exposure variable was the composite score of the VFQ-J11. The question items included in the VFQ-J11 were shown in S1 Table. The VFQ-J11 consists of 11 items reflecting the following seven domains: item 1 in general vision, item 8 to 10 in near vision, item 5 to 7 in distance vision, item 2 in dependency, item 11 in social functioning, item 3 in well-being/distress, and item 4 in role limitation. While item 1 was rated using a 6-point Likert scale, items 2 through 4 were rated using a 5-point Likert scale. Items 5 through 11 were rated with 6 choices, consisting of choices “1” to “5” of a 5-point Likert scale and choice “6” to indicate that the person does not perform the activity because of problems that are unrelated to vision[17]. If choice “6” was selected, the item was coded as “missing”[18]. Each item score was converted to a score between 0 and 100, with higher scores indicating better vision-specific QOL. The composite VFQ-J11 score was calculated as the average score of all items. For example, if the choice “6” was selected in items 2 through 4, which were coded as “missing”, the composite score was calculated as the average of the remaining 8 items.

### Outcomes

The primary outcome was frequent (≥2) falls over the preceding 1-month period, and the secondary outcome was any fall over the same period. To define fall history, the participants were asked the question, ‘Over the past year, have you fallen down?’, with the response options of ‘Yes’ or ‘No’. The participants who responded ‘Yes’ were then asked the following question: ‘How many times have you fallen down over the past month?’, with the response options of ‘Zero’, ‘Once’, ‘Twice’ or ‘Three or more times’. Those who responded at least ‘Twice’ were defined as experiencing the primary outcome. Those who responded at least ‘Once’ were defined as experiencing the secondary outcome.

### Statistical analysis

Baseline characteristics were presented using standard descriptive statistics: medians (interquartile ranges) for continuous variables and percentages for categorical variables. For the primary analysis, odds ratios (ORs) and 95% confidence intervals (95% CIs) for risk of fall were estimated using the multiple logistic regression model. In the logistic regression model, we adjusted for clinically relevant confounding factors: age, gender, presence of diabetes mellitus (defined as prescription of antidiabetic drug or HbA1c≥6.1%, as measured using the criteria of the Japanese Diabetes Society, which is equivalent to ≥6.5% as measured in the National Glycohemoglobin Standardization Program[19]) and hypertension (defined as systolic/diastolic blood pressure ≥140/90 mmHg or the prescription of antihypertensive medication), history of cerebrovascular disease (CVD), mental health (defined using mental health in the short form [SF]-36), living alone (defined using the self-reported questionnaire), and Timed Up and Go (TUG). Reportedly, mental health in the SF-36 is correlated with the Zung Self-rating Depression Scale and can be used to identify people with depressive symptoms in the general population of Japan[20]. The TUG test, which is considered to be a surrogate of functional mobility integrated with gait function and balance, is commonly used to assess the physical performance of older people[21]. A p-value of <0.05 was considered statistically significant. All analyses were performed with multiple imputation methods using chained equation by STATA version 14 (Stata Corp, College Station, TX, USA).

### Subscale analysis

We defined the subscale score in the VFQ-J11 as the average score within the same domains. For example, to calculate the subscale of near vision, which includes three items, the three item scores were added and divided by 3. We examined the association between each subscale and the outcome in separate logistic models which estimated ORs and 95% CIs for frequent falls and any fall.

### Sensitivity analysis

To minimize the selection bias caused by excluding participants who did not complete the VFQ-J11questionnaire, we conducted sensitivity analyses. We performed the analyses with multiple imputation for missing VFQ-J11 data among the participants, after excluding those younger than 65 years and those with outcome values missing. These sensitivity analyses were performed for the same comparisons and outcomes as the primary analyses.

## Results

### Baseline characteristics

**Fig 1:**
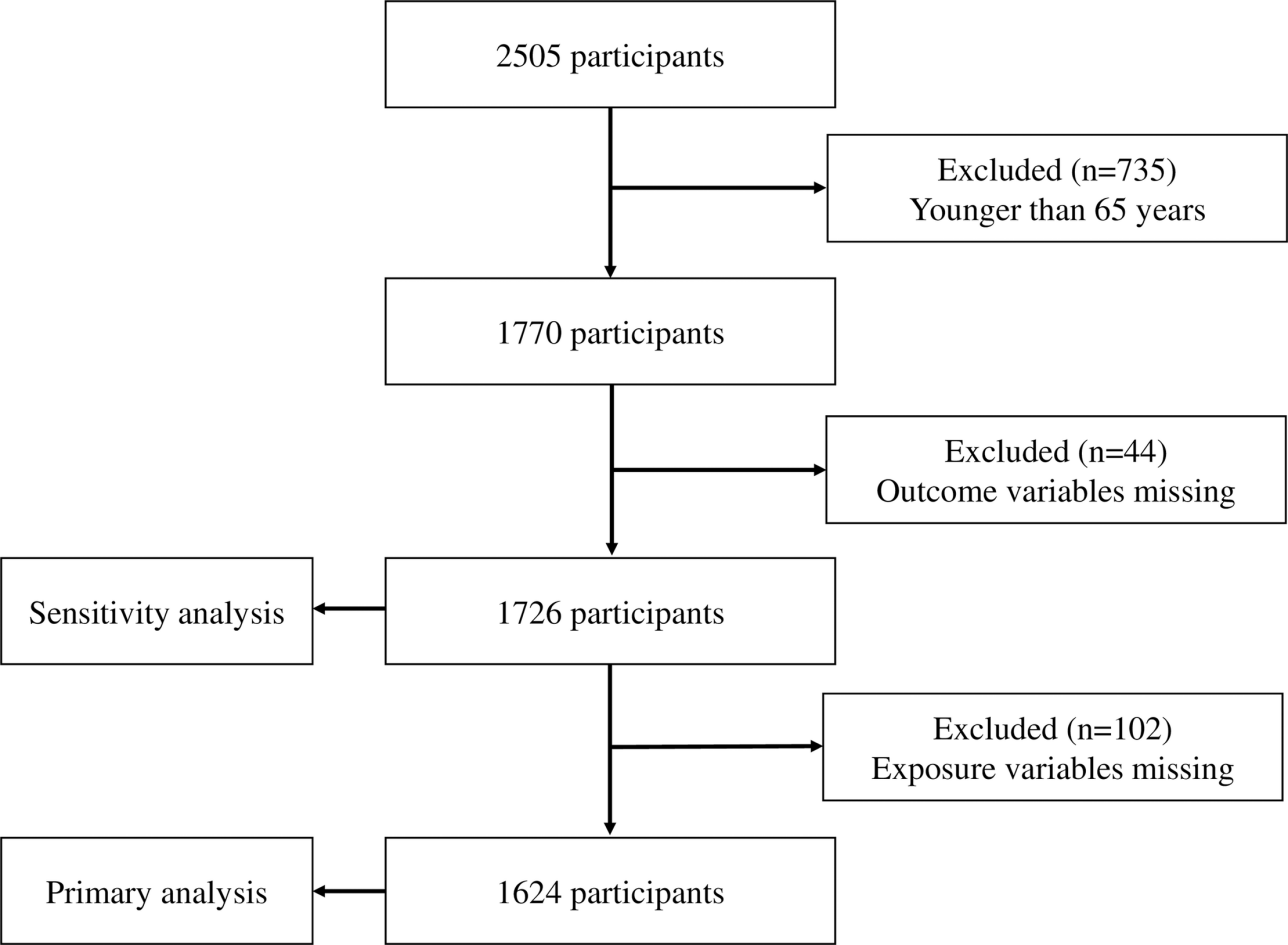
Selection of subjects.

LOHAS enrolled 2505 participants in 2010. We excluded those aged <65 years and those with missing data for the exposure and outcome variables, leaving 1624 for inclusion in the primary analysis (Fig 1). The reliability of this scale, assessed using Cronbach’s alpha, was 0.87, indicating good reliability. The baseline characteristics of the participants are summarized in Table 1. Median (interquartile range) age was 73 (69–77) years, and median (interquartile range) VFQ-J11 composite score was 86.8 (76.0–95.9). Any fall and frequent falls were reported by 13.9% and 5.4% of the participants in the primary analysis, respectively.

**Table 1:** Baseline characteristics.

	All participants (n = 1624)	No. of participants with missing data
Age (years)	73 (69–77)	0
Gender (%; male)	690 (42.5)	0
Hypertension (%)	1089 (71.4)	99
Diabetes (%)	121 (8.6)	212
History of CVD (%)	80 (5.1)	60
Mental health score in SF 36	75 (60–90)	79
Timed up & go (sec)	7.6 (6.5–8.9)	7
Living alone (%)	215 (17.3)	383
Number of falls		
Any falls	226 (13.9)	0
Frequent falls	87 (5.4)	0
VFQ-J11		
Composite score	86.8 (76.0–95.9)	0
General vision	80 (60–80)	0
Near vision	83.3 (75–100)	1
Distance vision	91.7 (75–100)	0
Dependency	100 (75–100)	0
Social functioning	75 (75–100)	2
Well-being/distress	100 (75–100)	0
Role limitation	100 (75–100)	0

Note: Values for categorical variables are given as a percentage; values for continuous variables are given as median (interquartile range). Abbreviations: CVD, cerebrovascular disease.

### Primary outcome

Frequent falls in the preceding month were experienced by 87 participants. We estimated ORs for frequent falls according to the VFQ-J11 composite score. Odds ratio in the logistic models are shown in Table 2. The logistic models showed that VFQ-J11composite score was significantly associated with frequent falls (OR per 10 points, 0.80; 95% CI, 0.68–0.93).

**Table 2:** Association of VFQ-J11 with frequent falls.

	Crude		Adjusted	
	OR	95% CI	OR	95% CI
VFQ-J11 composite score per 10 points	0.73	0.63–0.85	0.80	0.68–0.93

Note: Odds ratios were adjusted by age, gender, presence of diabetes and hypertension, history of CVD, mental health, TUG and living alone.

### Secondary outcome

Any fall in the preceding month was experienced by 226 participants. We estimated the ORs for any fall according to the VFQ composite score. The logistic models showed that VFQ composite score was significantly associated with any fall as well as frequent falls (OR per 10 points, 0.84; 95% CI, 0.76–0.94) (Table 3).

**Table 3:** Association of VFQ-J11 with any fall.

	Crude		Adjusted	
	OR	95% CI	OR	95% CI
VFQ-J11 composite score per 10 points	0.78	0.70–0.86	0.84	0.76–0.94

Note: Odds ratios were adjusted by age, gender, presence of diabetes and hypertension, history of CVD, mental health, timed up & go and living alone.

### Subscale analysis

We examined the association of VFQ-J11 subscale scores with frequent falls and any fall in separate logistic models. The ORs are shown in S2 Table. Near vision, well-being/distress, and role limitation were significantly associated with both frequent falls and any fall. On the other hand, distance vision was not significantly associated with frequent falls or any fall.

### Sensitivity analysis

We selected 1726 participants for the sensitivity analyses (Fig 1). The comparison of baseline characteristics between those included in the primary analysis and those included in sensitivity analysis is shown in S3 Table. The proportion of males among the participants in the sensitivity analysis was 41.6%, which was slightly less than among those in the primary analysis. For the primary outcome and secondary outcome, the ORs are shown in S4 Table. ORs for the subscale analysis are shown in S5 Table. Similar to the primary analysis, the composite score of VFQ-J1, near vision, well-being/distress, and role limitation were significantly associated with both frequent falls and any fall.

## Discussion

This is the first study to investigate the association of VFQ-J11 score with falls. We found significant associations of the VFQ-J11 with both any fall and frequent falls in the previous month. We also found significant associations of three VFQ-J11 subscales (near vision, well-being/distress, and role limitation) with both any fall and frequent falls in the previous month.

There are several possible reasons why the VFQ-J11 composite score is associated with falls in community-dwelling older adults. First, the VFQ-J11 is highly associated with objective parameters of visual function, such as visual acuity and field of vision[13]. The VFQ-J11 was developed using item-response theory, which selected items having high discriminative ability for visual function based on the slope parameter and the location parameter. This method provided the VFQ-J-11 with superior criterion validity to the VFQ-25. Second, the VFQ-J11 evaluates visual function from several different aspects using 7 domains: general vision, near vision, distance vision, dependency, social functioning, well-being/distress, and role limitation. A number of studies have evaluated the association between self-reported visual function and falls using a single aspect of vision, such as distance vision or near vision [8,10,11], although results have been inconsistent. According to our subscale analysis, we found that the association between each domain in the VFQ-J11 and falls differed, and that the OR per 10 points of VFQ-J11 composite score was lower than the ORs of the individual subscales, which suggests that evaluation using a single aspect of self-reported visual function is not sufficient to detect the association with falls. Third, we excluded participants younger than 65 years from the study population. Jaffee EG et al reported that insufficient vison was significantly associated with post-discharge falls among the participants aged ≥65 years but not among those aged <65 years[22]. The association of vision-specific QOL with falls could be stronger among older adults than younger adults.

According to the subscale analysis, near vision, well-being/distress, and role limitation were associated with both frequent falls and any fall. On the other hand, distant vision was not associated with either frequent falls or any fall. The association with well-being/distress and role limitation was consistent with the results of the development of the VFQ-J11[13]. The question items of well-being/distress and role limitation had negative location parameters, indicating that individuals with impaired vision find it difficult to correctly answer these questions; these items also had higher slope parameters, indicating good discrimination for respondent’s latent traits. Although the other subscale items, including near vision, had various slope and location parameters, near vision was associated with falls. Impairment of near vision could be a reflection of impairment of depth perception, which was reported to be associated with falls[3,23,24]. Individuals with impaired near vision may have difficulty avoiding a blockade on the ground, which could lead to falls. However, whether the subscale of near vision is associated with depth perception remains unclear, and further investigation is needed.

The present study has several strengths. First, it is the first study to investigate the association between falls and vision-specific QOL retrieved using the validated VFQ-J11 questionnaire. The VFQ-J11 was developed using item response theory and is reported to be more strongly associated with aspects of visual function, such as visual acuity and field of vision, than the VFQ-25[13]. Second, the VFQ-J11 evaluates visual function using multiple domains, enabling us to investigate the association between falls and visual function in the self-reported questionnaire from several aspects. Previous reports have investigated the association between falls and self-reported vision only in terms of limited visual functions, such as distance vision or near vision[8,10,11]. Third, this is the first study to investigate the association between the VFQ-J11 and an important clinical outcome in general population. As the VFQ-J11 was developed from a specific population of outpatients in departments of ophthalmology recruited using convenience sampling[12,13], it has been unclear how the VFQ-J11 is associated with clinical outcomes in the general population. Fourth, we adjusted for potential confounding factors, such as mental health and functional mobility, that might affect the association between falls and VFQ-J11 score. We could not evaluate the effect of other relevant problems, such as delirium, which are reported to be associated with visual impairment in hospitalized older patients[25]; however, the population in this study, which was recruited using a population-based method, was so different from hospitalized patients that it is unlikely that participants with delirium could be recruited and answer the questionnaire in this study. Recently, functional disability was reported as a potential mediator of the association between vision impairment and fall risk, indicating that the direct effect of poor vision on fall risk was not significant[26]. In the present study, the VFQ-J11 composite score was significantly associated with any fall and frequent falls, even after adjusting for TUG, a surrogate index of mobility, indicating that self-reported vision function itself might have a direct impact on risk of falls.

We feel that the findings in this present study have important implications from a clinical and public-health perspective. Although it is already known that objective vision metrics, such as visual acuity and visual field, are associated with falls, actual measurement of these items need special settings and have associated costs, so implementation in all clinical or public-health settings could be difficult. Detecting patients or residents with poor vision and risk of falls using a self-administered questionnaire could be easily implemented in several settings. For example, public-health sectors, such as government institutions, could send their residents the VFQ-J11 questionnaire by mail to effectively detect those with impaired vision-specific QOL and refer them to ophthalmologists.

However, the study also has several limitations. First, because of its cross-sectional design, reverse causality was possible. While impaired vision-specific QOL is considered as a plausible cause of falls, reverse causality could be possible in the association of fall with several individual domains of vision-related QOL, such as mental health and functional limitation. Second, the association of vision-specific QOL with falls could not be adjusted by the presence of dementia, which might be a potential confounding factor. Reportedly, individuals with dementia are likely to have difficultly completing self-reported questionnaires by themselves[27]. This study included participants who filled in all questions on the VFQ-J11, suggesting that included participants may be less likely to have dementia than a sample that included subjects who could not complete the VFQ-J11 and that the results of this study might have been only minimally influenced by this confounding factor. Third, some confounding variables included missing values, which could lead to selection bias. However, we conducted multiple imputation for these variables to mitigate the bias. Furthermore, we conducted sensitivity analysis with multiple imputation for missing values on the VFQ-J11. The results of sensitivity analyses were similar to the primary analysis, supporting the robustness of the results of the present study. Fourth, the primary outcome measure was self-reported falls over the preceding one-month period. Although a one-month period could be too short to accurately discriminate participants with fall history from those without it, questions assessing falls over longer periods could lead to recall biases because participants could be able to remember their history of falls less precisely over longer periods of time. To minimize these biases, we evaluated the association of VFQ-J11 with both frequent falls and any fall and found the similar associations with both outcomes. Fifth, objective vison parameters, such as visual acuity and visual field, were not measured in this study, and the association of the VFQ-J11 with those objective parameters in the population in this study was unknown. However, VFQ-J11 has been validated using visual acuity and visual field in a previous study[13], which showed that the composite score of VFQ-J11 was associated with those parameters more than VFQ25. Finally, although the composite score of VFQ-J11 was validated, the subscales of VFQ-J11 have not been validated. However, the method to compute them was the same as is used for the VFQ25[18] and the subscales themselves are associated with both frequent falls and any fall, such as near vision, well-being/distress, and role limitation, and could be useful to identify older adults with poor vision-specific QOL and risk of falls.

In conclusion, we found that the composite score of the VFQ-J11 was associated with falls. We may consider adding self-reported visual function and vision-specific QOL to conventional risk factors for fall among older adults. This finding has important clinical implications. First, using the self-administered VFQ-J11 questionnaire, we could detect individuals with visual impairment associated with falls. This might in turn lead to population-based interventions to modify visual impairment to prevent falls. Second, improvement in VFQ-J11 scores (e.g., after cataract surgery) might prevent falls. However, further longitudinal studies are needed to investigate the association between change in VFQ-J11 score and falls.

## Supporting information

S1 Table Question items of the VFQ-J11. (DOCX)

S2 Table Association of VFQ-J11 subscale with frequent falls and any fall. (DOCX)

S3 Table Comparison of baseline characteristics of participants in final analysis and those in sensitivity analysis. (DOCX)

S4 Table Association of VFQ-J11 with frequent falls and any fall in sensitivity analysis. (DOCX)

S5 Table Association of VFQ-J11 subscale with frequent falls and any fall in sensitivity analysis. (DOCX)
