# Supplementary material for: Association between vision-specific quality of life and falls in community-dwelling older adults: LOHAS
Source: PLoS One. 2018 Apr 24;13(4):e0195806. doi: 10.1371/journal.pone.0195806 (PMC5978984; doi:10.1371/journal.pone.0195806)
Supplement: S1 Table — (DOCX) [file pone.0195806.s001.docx]

**S1 Table. Question items of the VFQ-J11**

|  | **VFQ-J11 items** | **Domains** |
| --- | --- | --- |
| #1. | At the present time, would you say your eyesight using both eyes (with glasses, or contact lenses, if you wear them) is excellent, good, fair, poor or very poor or are you completely blind? | General vision |
| #2. | I need a lot of help from others because of my eyesight. | Dependency |
| #3. | I worry about doing things that will embarrass myself or others, because of my eyesight. | Well-being/Distress |
| #4. | Do you accomplish less than you would like because of your vision? | Role limitation |
| #5 | Because of your eyesight, how much difficulty do you have recognizing people you know across a room? | Distance vision |
| #6. | Because of your eyesight, how much difficulty do you have going out to see movies, plays, or sports events? | Distance vision |
| #7. | How much difficulty do you have reading street signs or the names of stores? | Distance vision |
| #8. | How much difficulty do you have reading ordinary print in newspapers? | Near vision |
| #9 | How much difficulty do you have work or hobbies that require to see well up close, such as cooking, sewing, fixing things around the house, or using hand tools? | Near vision |
| #10 | Because of your eyesight, how much difficulty do you have finding something on a crowded shelf? | Near vision |
| #11 | Because of your eyesight, how much difficulty do you have seeing how people react to things you say? | Social functioning |
