# Supplementary material for: Association between vision-specific quality of life and falls in community-dwelling older adults: LOHAS
Source: PLoS One. 2018 Apr 24;13(4):e0195806. doi: 10.1371/journal.pone.0195806 (PMC5978984; doi:10.1371/journal.pone.0195806)
Supplement: S3 Table — (DOCX) [file pone.0195806.s003.docx]

**S3 Table. Comparison of baseline characteristics** **of participants in final analysis and those in sensitivity analysis**

|  | All participants  in final analyses  (n= 1624) | All participants  in sensitivity analyses  (n=1726) |
| --- | --- | --- |
| Age (years) | 73 (69-77) | 73 (69- 77) |
| Gender (%; male) | 690 (42.5) | 718 (41.6) |
| Hypertension (%) | 1089 (71.4) | 1163 (71.8) |
| Diabetes (%) | 121 (8.6) | 129 (8.6) |
| History of CVD (%) | 80 (5.1) | 85 (5.1) |
| Mental health score in SF 36 | 75 (60-90) | 75 (60- 90) |
| Timed up & go (sec) | 7.6 (6.5-8.9) | 7.6 (6.6- 8.9) |
| Living alone (%) | 215 (17.3) | 227 (17.4) |
| Number of falls |  |  |
| Any falls | 226 (13.9) | 241 (14.0) |
| Frequent falls | 87 (5.4) | 92 (5.3) |
