# Supplementary material for: Association between vision-specific quality of life and falls in community-dwelling older adults: LOHAS
Source: PLoS One. 2018 Apr 24;13(4):e0195806. doi: 10.1371/journal.pone.0195806 (PMC5978984; doi:10.1371/journal.pone.0195806)
Supplement: S4 Table — (DOCX) [file pone.0195806.s004.docx]

**S4 Table.** **Association of VFQ-J11 with frequent falls and any fall in sensitivity analysis**

|  | Crude | | Adjusted | |
| --- | --- | --- | --- | --- |
|  | OR | 95% CI | OR | 95% CI |
| Frequent falls |  |  |  |  |
| VFQ-J11 composite score  per 10 points | 0.74 | 0.64- 0.86 | 0.81 | 0.69- 0.94 |
| Any fall |  |  |  |  |
| VFQ-J11 composite score  per 10 points | 0.78 | 0.71- 0.86 | 0.85 | 0.77- 0.95 |
