# Supplementary material for: Association between vision-specific quality of life and falls in community-dwelling older adults: LOHAS
Source: PLoS One. 2018 Apr 24;13(4):e0195806. doi: 10.1371/journal.pone.0195806 (PMC5978984; doi:10.1371/journal.pone.0195806)
Supplement: S5 Table — (DOCX) [file pone.0195806.s005.docx]

**S5 Table.** **Association of VFQ-J11 subscale with frequent falls and any fall in sensitivity analysis**

|  | ORs | 95% CI |
| --- | --- | --- |
| Frequent falls |  |  |
| General vision | 0.88 | 0.78- 1.002 |
| Near vision | 0.88 | 0.78- 0.99 |
| Distance vision | 0.95 | 0.84- 1.08 |
| Dependency | 0.94 | 0.86- 1.02 |
| Social functioning | 0.92 | 0.82- 1.03 |
| Well-being/distress | 0.92 | 0.86- 0.997 |
| Role limitation | 0.84 | 0.77- 0.92 |
| Any fall |  |  |
| General vision | 0.93 | 0.85- 1.01 |
| Near vision | 0.91 | 0.84- 0.99 |
| Distance vision | 0.97 | 0.88- 1.06 |
| Dependency | 0.94 | 0.89- 0.99 |
| Social functioning | 0.92 | 0.86- 0.999 |
| Well-being/distress | 0.94 | 0.89- 0.99 |
| Role limitation | 0.89 | 0.84- 0.95 |
